# Genome survey sequencing for the characterization of genetic background of *Dracaena cambodiana* and its defense response during dragon’s blood formation

**DOI:** 10.1371/journal.pone.0209258

**Published:** 2018-12-14

**Authors:** Xupo Ding, Wenli Mei, Shengzhuo Huang, Hui Wang, Jiahong Zhu, Wei Hu, Zehong Ding, Weiwei Tie, Shiqing Peng, Haofu Dai

**Affiliations:** 1 Key Laboratory of Biology and Genetic Resources of Tropical Crops of Ministry of Agriculture and Rural Affairs, Institute of Tropical Bioscience and Biotechnology, Chinese Academy of Tropical Agricultural Sciences, Haikou, Hainan, People’s Republic of China; 2 Hainan Key Laboratory for Research and Development of Natural Products from *Li* folk Medicine, Institute of Tropical Bioscience and Biotechnology, Chinese Academy of Tropical Agricultural Sciences, Haikou, Hainan, People’s Republic of China; German Cancer Research Center (DKFZ), GERMANY

## Abstract

Dragon’s blood collected from the genus *Dracaena* is used as a renowned traditional medicine in various cultures worldwide. However, the genetics of the genus *Dracaena* and the formation mechanism of dragon’s blood remain poorly understood. Here, we generate the first draft genome reference assembly of an elite Chinese *Dracaena* species, *Dracaena cambodiana*, from next-generation sequencing data with 89.46× coverage. The reads were assembled into 2,640,704 contigs with an N50 length of 1.87 kb, and a 1.05 Gb assembly was finally assembled with 2,379,659 scaffolds. Furthermore, 97.75% of the 267,243 simple sequence repeats identified from these scaffolds were mononucleotide, dinucleotide, and trinucleotide repeats. Among all 53,700 predicted genes, 158 genes involved in cell wall and plant hormone synthesis and reactive oxygen species scavenging showed altered regulation during the formation of dragon’s blood. This study provides a genomic characterization of *D*. *cambodiana* and improves understanding of the molecular mechanism of dragon’s blood formation. This report represents the first genome-wide characterization of a *Dracaena* species in the Asparagaceae.

## Introduction

Asparagaceae is a new family derived from the Liliaceae by the Angiosperm Phylogeny Group (APG) in 1998 [1]. In this family, *Dracaena* is one of the oldest genera, and *Dracaena* species are used as ornamental or horticultural plants worldwide [2, 3]. An injured trunk or branch of a *Dracaena* plant can exude a red resin, known as dragon’s blood, which has been utilized as a traditional medicine for wounds, fractures, piles, leucorrhea, diarrhea, stomach and intestinal ulcers, and even some types of cancer in the histories of many cultures [4–8]. Modern chemical and pharmacological studies have indicated that the flavonoids, saponins, terpenes, and steroids in dragon’s blood are pharmacodynamic compounds [9–11]. In China and Southeast Asian countries, *D*. *cambodiana* has been preferred for dragon’s blood extraction and is widely cultivated [12]. Due to their medicinal and economic importance, wild *Dracaena* plants have been exploited excessively, and many of them, including *D*. *cambodiana*, have been considered endangered [13].

The dragon’s blood product is pharmaceutically valuable, and its availability is limited by the exhaustion of its plant sources and its time-consuming preparation. To date, the molecular mechanism of dragon’s blood formation has remained unclear, though it is thought to be a defensive metabolite secreted from the wounded stems of *Dracaena* for protection against pathogens [4, 14]. This hypothesis suggests that the formation of dragon’s blood involves a special defense response in *Dracaena* plants. The expression of defense-related genes and the synthesis of defensive substances are two crucial defense mechanisms protecting plants from biotic stress [15], and these strategies include the NBS-LRR genes and the endogenous plant hormone salicylic acid [16, 17]. However, the limited genomic and genetic resources available for *Dracaena* impede the mechanistic exploration of dragon’s blood formation. Only the expression of genes related to flavonoid and saponin synthesis during dragon’s blood formation has been described through transcriptome sequencing [18, 19].

Next-generation sequencing (NGS) has facilitated plant genomic research in the past ten years. More than a hundred plant species, including several medicinal plants, such as *Cannabis sativa* [20], *Gastrodia elata* [21], *Dendrobium officinale* [22], *Salvia miltiorrhiza* [23], and *Panax notoginseng* [24], have been successfully sequenced using NGS technologies. Some other medicinal plants, such as *P*. *ginseng* and *Eucommia ulmoides*, have also been subjected to genome sequencing [25, 26].

In this study, we sequenced the genome of *D*. *cambodiana* and performed a draft assembly to examine the genetic background of *Dracaena* and the molecular mechanism of dragon’s blood formation. Understanding this special defense response of *Dracaena* will allow new advancements in molecular breeding for this important medicinal plant.

## Materials and methods

### Plant materials and DNA extraction

Five tender branches about 10-20cm were collected from individual *D*.*cambodiana* plant on the Dazhou Island (Wanning, Hainan Province, China) after authorized by the operator on duty, and then were planted in plantation at the Institute of Tropical Bioscience and Biotechnology, Haikou, China (19° 59′ N, 110° 19′ E). Both original *Dracaena* tree on the Dazhou Island and its five branches cultured are all still being alive for now. Leaves sample collecting have been authorized by our institute within the project supported by funding of 1630052016002. The young leaves of *D*. *cambodiana* were disinfected with 75% ethyl alcohol and then frozen and stored in liquid nitrogen for genomic DNA extraction. The total genomic DNA of *D*. *cambodiana* was extracted using a plant genomic DNA extraction kit (Tiangen Biotech, Beijing, China). Subsequently, its quantity and quality were assessed using a ScanDrop 100 spectrophotometer (Analytik Jena, Germany) and 1.5% agarose gel electrophoresis.

### Genome sequencing and genomic size estimation

Paired-end (PE) libraries with insert sizes of 270 and 500 base pairs (bp) were constructed following the Illumina standard protocol [27]. Sequence data were generated using the Illumina HiSeq 2500 platform. The filtered clean reads were used for estimation of genome size, percentage of repetitive sequence, and heterozygosity by using k-mer analysis [28].

### Genomic sequence assembly and GC content estimation

The preprocessed PE reads were assembled using SOAPdenovo, and the optimal k-mer size was selected for the maximum N50 of contigs [29]. The scaffolds were progressively constructed with PE reads of different insert sizes. Only scaffolds of more than 1000 bp in length were retained in the final assembly. The GC content was calculated using 10 kb nonoverlapping sliding windows along the assembled sequence.

### Simple sequence repeats (SSRs)

To inspect the complement of SSRs and provide strategies for the genome sequencing or assembly of *D*. *cambodiana*, an appropriate repetitive sequence library was constructed for predicting repeat sequences using LTR_FINDER with the *de novo* data described earlier [30]. Then, TRE and RepeatMasker 3.3.0 were used to search for homologous tandem or interspersed repeats, respectively [31, 32]. SSR motifs were determined using SciRoKo [33].

### Gene prediction and annotation

GeneID was used for *de novo* gene prediction [34] and corrected with the previous transcripts data [18], and functional annotation was performed with Kyoto Encyclopedia of Gene and Genomes (KEGG) [35], Gene Ontology (GO) [36], Clusters of Orthologous Groups (COGs) [37], euKaryotic Clusters of Orthologous Groups (KOGs) [38], TrEMBL [39], Swiss-Prot [39], Pfam [40], Nt and Nr [41]. For gene family identification, the putative genes of *D*. *cambodiana* were clustered using OrthoMCL [42] with the unigenes of *Arabidopsis thaliana* (TAIR10), *Asparagus officinalis* (Asrof.V1), *Dendrobium officinale* (ASM160598v1), *Populus euphratica* (PopEup_1.0), and *Picea glauca* (PG29_V4.1). These sequences were downloaded from public databases. The Venn diagram was generated in R for numbering the gene cluster [43].

### Genes involved in defense response during dragon’s blood formation in *D*. *cambodiana*

Defense genes encoding lipoxygenase (LOX), allene oxide synthase (AOS), allene oxide cyclase (AOC), 12-oxo-phytodienoic acid reductase (OPAR), phenylalanine ammonia lyase (PAL), isochorismate synthase 1 (ICS1), acyl-adenylate/thioester-forming enzyme (PBS3), BAHD acyltransferase (EPS1), proline dehydrogenase (PDG), trehalose phosphate synthase (TPS), ascorbate peroxidase (APX), glutathione reductase (GR), superoxide dismutase (SOD), peroxidase (POD), P450, and their related proteins that are involved in jasmonic acid, salicylic acid, proline, and trehalose synthesis or reactive oxygen species (ROS) response during dragon’s blood formation in *D*. *cambodiana* were selected from the gene prediction results and then illuminated by using HemI 1.0 [44] with our published transcriptome database. The genes encoding the enzymes that catalyze the synthesis of cell wall components in *D*. *cambodiana* were also analyzed using the same methods. The RNA-seq samples were collected from 6 cm above the injection site in 3-year-old *D*. *cambodiana* stems at 3 days and 6 days after injection with a special inducer [18]. Healthy stems cut from *D*. *cambodiana* trees were used to generate material for the 0-day library.

### Quantitative rela-time RT-PCR

To validate the RNA-seq results, quantitative RT-PCR was conducted by the following method. The product cDNA was diluted into 50 ng·μL^-1^, and 1μL was used for each Real-time quantitative PCR (RT-qPCR). The RT-qPCR reaction mixtures (20μL) also contained 0.4 μL of each gene specific primer, 10μL 2×TransStart qPCR SuperMix (Transgen, Beijing, China) and 8.2μL RNase-free water. The RT-qPCR thermal cycling included cDNA degenerated in 94°C for 30 sec, with 40 cycles of 94°C for 5 sec and then 60°C for 30 sec in the Strata Gene Mx3005P Real-Time PCR System (Agilent lnc., USA) with the SYBR green method. The *β-actin* of *D*. *cambodiana* was chosen as a housekeeping gene with internal control their relative expression were assessed with 2^-ΔΔCt^ method [18]. All RT-qPCR experiments were performed in triplicate and the gene-specific primers used in expression analysis are listed in S1 Table.

## Results

### Genome sequencing and genome size estimation

**Fig 1:**
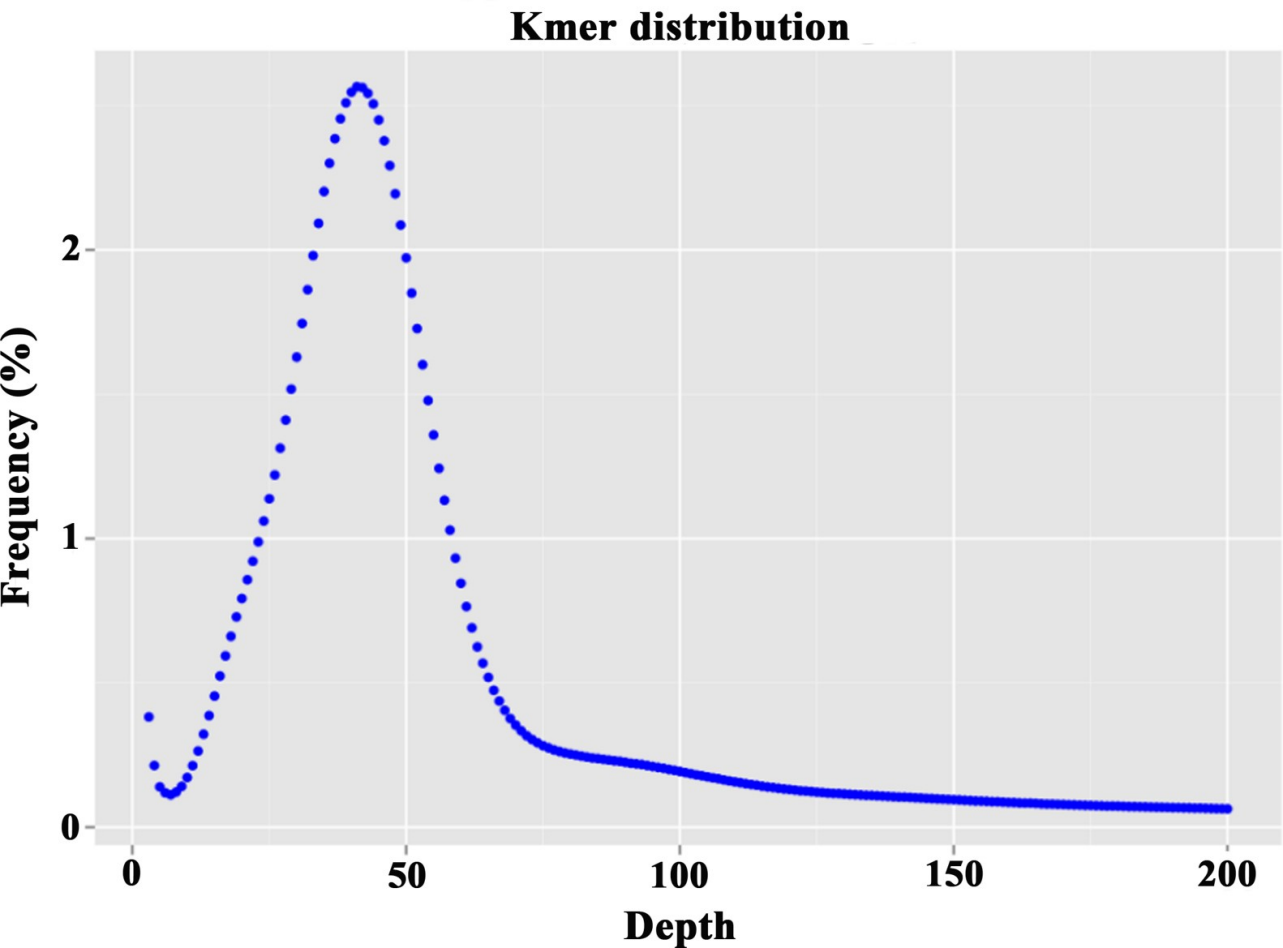
K-mer (k = 21) analysis for estimating the size of the *D*. *cambodiana* genome. The occurrence of 21-mers was calculated using Jellyfish version 2.1.3, based on the sequencing data from three short-insert libraries of *D*. *cambodiana*. The genome size was estimated by the following formula: Genome size = K-mer num/Peak depth. The subpeak on the left of the main peak was caused by genome heterozygosity.

To accurately investigate the genomic background of *D*. *cambodiana*, three libraries with insert lengths of 270 and 500 bp were constructed. The Q20 or Q30 values for evaluating the base quality of the sequencing data were above 90%. On the basis of these data, a total of 100.22 Gb raw sequencing data were provided, and the sequence coverage was approximately 89.46×. (Table 1). Then, these clean data were used for k-mer analysis. For the 21-mer frequency distributions, the peak of depth distribution was approximately 73, and the estimated genome size was 1.12 Gb (Fig 1). Similarly, the ratios of repetitive sequences and heterozygosis were calculated using the k-mer distribution, with results of 53.45% and 0.38%, respectively.

**Table 1:** Statistics of sequencing data.

Library	Data (Gb)	Depth (×)	Q20 (%)	Q30 (%)	Genome Size (Gb)	Heterozygous Ratio (%)	Repeat (%)	GC Content (%)
270 bp (BMK)	57.24	51.10	96.03	90.81	1.12	0.42	55.53	39.04
270 bp (BGI)	15.52	13.85	95.36	90.40	1.17	0.60	64.47	37.79
500 bp (BGI)	27.46	24.51	96.57	93.11	1.12	0.78	68.52	38.33
Total	100.22	89.47	-	-	1.12	0.38	53.45	37.35

### Genome sequence assembly and GC content analysis

**Fig 2:**
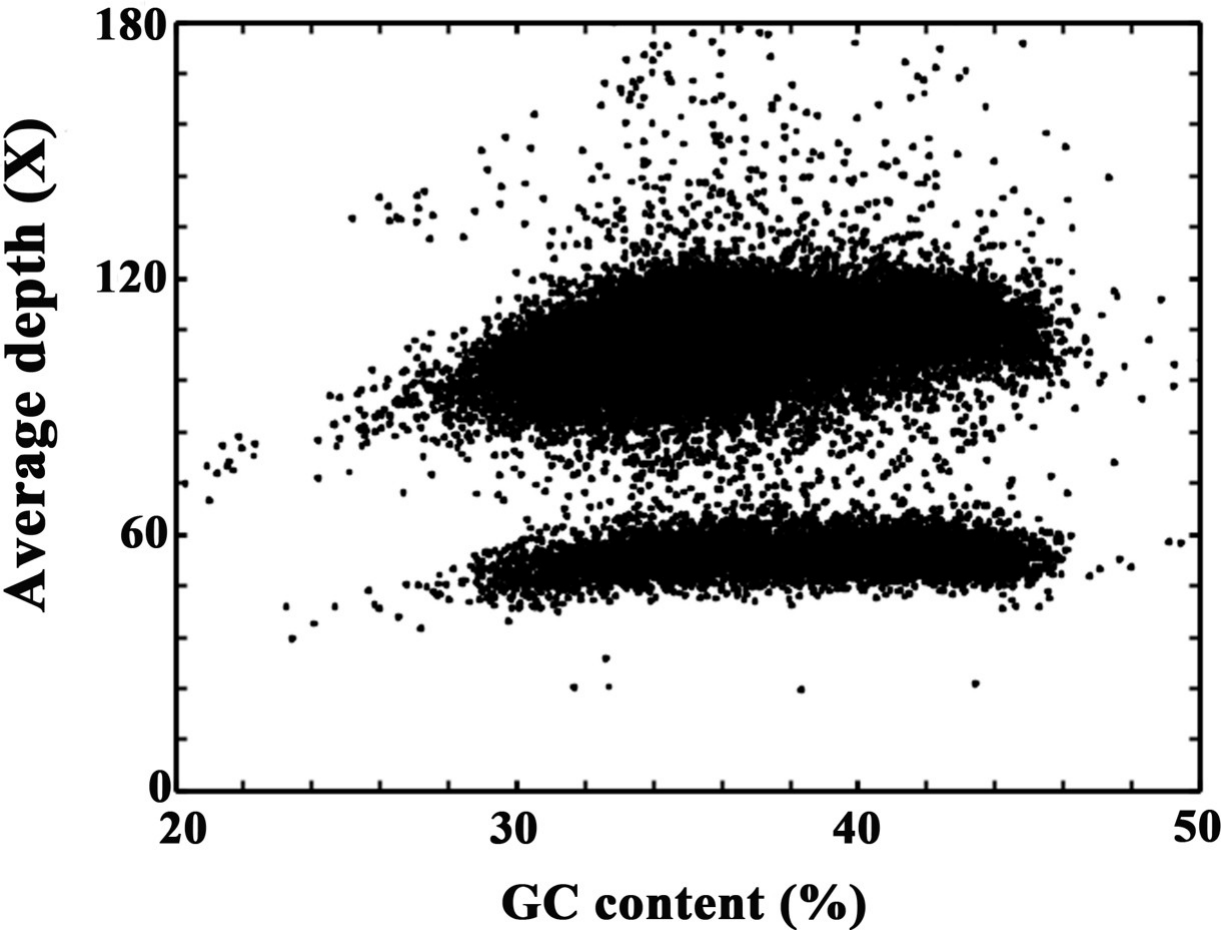
GC content and average sequencing depth of the sequencing data used for assembly. The x-axis represents GC content **(**percent) across every 10-kb nonoverlapping sliding window.

The N50 of the contigs was 1.87 kb, with a total length of 1.01 Gb, and the N50 of the scaffolds was 3.19 kb, with a total length of 1.06 Gb (Table 2). The longest contig and scaffold were 139,994 bp and 348,119 bp, respectively. The GC content of the *D*. *cambodiana* genome was 37.35%, which is considered a moderate GC content (Fig 2). Moreover, the GC depth distribution was obviously divided into two peaks. This result was partly caused by a 0.38% heterozygosity rate (Fig 2).

**Table 2:** Statistics of the assembled genome sequences.

Contigs	Size (bp)	Number
N90	127	1,530,375
N80	247	718,498
N70	601	380,997
N60	1,094	217,665
N50	1,859	124,743
Longest	139,994	
Total Size	1,014,907,800	
Total Number (>100 bp)	2,640,704	
Total Number (>1000 bp)	237,871	
Total Number (>2000 bp)	115,120	
**Scaffolds**		
N90	127	1,232,630
N80	349	484,711
N70	876	242,856
N60	1,710	130,180
N50	3,194	71,044
Longest	348,119	
Total Size	1,064,434,799	
Total Number (>100 bp)	2,379,659	
Total Number (>1000 bp)	215,426	
Total Number (>2000 bp)	112,153	
A	353,230,199	
T	343,143,868	
G	205,677,721	
C	210,744,899	
N	75,583,030	
Total (AGCT)	1,112,796,687	
G+C% (AGCT)	37.35	

### SSRs

**Fig 3:**
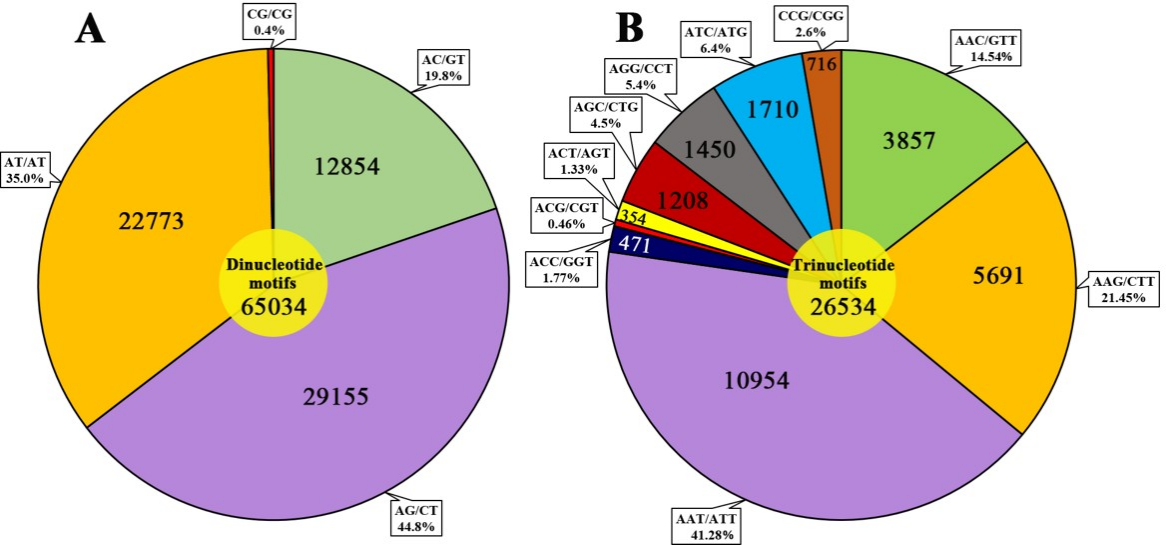
Percentages of various dinucleotide and trinucleotide repeat motifs in the *D*. *cambodiana* genome. A: Percentage of various dinucleotide repeat motifs in the *D*. *cambodiana* genome; B: Percentage of various trinucleotide repeat motifs in the *D*. *cambodiana* genome.

A total of 26,243 SSRs were identified from the draft assembly in 80,584 (30.15%) scaffolds (Table 3). Mononucleotides (63.48%), dinucleotides (24.34%), and trinucleotides (9.93%) comprised nearly 98% of the SSRs, and tetranucleotides, pentanucleotides, or hexanucleotides comprised only a small portion of the SSRs in our assembly (Table 3). Moreover, 208 types of motif were identified, including 2 types of mononucleotide, 4 types of dinucleotide, 10 types of trinucleotide, 31 types of tetranucleotide, 55 types of pentanucleotide, and 106 types of hexanucleotide repeats (S2 Table). Among the dinucleotide repeats, the common motifs were AG/CT (44.8%) and AT/AT (35.0%), followed by AC/GT (19.8%). The CG/CG motif was the least frequent among the detected dinucleotides (Fig 3A). Among the trinucleotide repeat motifs, the AAT/ATT motif was the most abundant, accounting for 41.28%, followed by AAG/CTT and AAC/GTT, accounting for 21.45% and 14.54%, respectively (Fig 3B).

**Table 3:** Simple sequence repeat types in *D*. *cambodiana* genome sequences.

Identifying Item	Number	Ratio
Total number of sequences examined	157,700	
Total size of examined sequences (bp)	692,500,389	
Total number of identified SSRs	267,243	100%
Number of SSR-containing sequences	80,584	30.15%
Number of sequences containing more than 1 SSR	49,908	18.68%
Number of SSRs present in compound formation	37,059	13.87%
Mononucleotide	169,646	63.48%
Dinucleotide	65,034	24.34%
Trinucleotide	26,534	9.93%
Tetranucleotide	4,817	1.80%
Pentanucleotide	849	0.32%
Hexanucleotide	363	0.14%

The combined results of TRE and RepeatMasker demonstrated that transposable elements (TEs) occupied 39.96% of the *D*. *cambodiana* genome, with 37.11% retroelements and 2.85% DNA transposons. Long terminal repeats (LTRs) were particularly abundant among the retroelements and accounted for 26.13% of the genome. In particular, LTR/Gypsy elements constituted 22.31% of the genome (Table 4).

**Table 4:** Statistics of transposable elements in *D*. *cambodiana* genome sequences.

Type		Number	Length	Rate
Class I	Unknown	10,604	2,894,506	0.20%
	DIRS	98,513	35,674,592	2.42%
	LARD	249,959	69,755,985	4.73%
	LINE	14,333	4,085,374	0.28%
	LINE/I	24	1,717	0.00%
	LINE/Jockey	35	2,158	0.00%
	LINE/L1	38,968	11,114,055	0.75%
	LINE/R2	102	5,196	0.00%
	LINE/RTE	74,746	22,500,966	1.53%
	LTR	20,581	5,524,380	0.37%
	LTR/Copia	146,843	43,854,672	2.97%
	LTR/ERV	4	208	0.00%
	LTR/Gypsy	1,011,391	328,934,978	22.31%
	LTR/Retrovirus	20,344	7,119,781	0.48%
	PLE	1,823	1,83,510	0.01%
	SINE	34,851	5,707,367	0.39%
	TRIM	43,794	9,911,671	0.67%
Class II	Unknown	5,301	551,987	0.04%
	Academ	6	323	0.00%
	Ginger2	1	145	0.00%
	ISL2EU	12	613	0.00%
	Kolobok	1,395	111,550	0.01%
	MuDR	14,363	2,915,206	0.20%
	Novosib	980	114,432	0.01%
	Sola	43	2,830	0.00%
	Crypton	24	1,719	0.00%
	Helitron	4,880	564,481	0.04%
	MITE	41,734	5,968,723	0.40%
	Maverick	184	46,804	0.00%
	TIR	54,052	16,426,219	1.11%
	TIR/CACTA	13,151	1,653,451	0.11%
	TIR/Mutator	1	78	0.00%
	TIR/P	310	21,580	0.00%
	TIR/PIF-Harbinger	4,860	402,212	0.03%
	TIR/PiggyBac	38	2143	0.00%
	TIR/Tc1-Mariner	113	10,799	0.00%
	TIR/hAT	52,306	13,205,329	0.90%
	Potential host gene	13,431	3,048,967	0.21%
	Unknown	1,583,577	265,683,012	18.02%
	Total without overlap	3,619,823	700,172,467	47.49%

### Protein-encoding gene prediction and annotation

We predicted 53,700 genes by using Gene ID (Table 5). The average lengths of the identified genes, exons, and introns were 2,030.67, 197.91, and 636.37 bp, respectively (Table 5). 44.56%of the gene predicated were interactively supported by the transcripts in public RNA-seq (S3 Table). Of these 53,700 predicted genes in the *D*. *cambodiana* assembly, 38,162 mapped genes were known genes in the public databases, of which 36,901 genes had Nt homologs, 22,153 had TrEMBL homologs, 14,873 had Swiss-Prot homologs, 12,859 had Pfam homologs, 13,093 had KOG homologs, 12,510 had GO homologs, 9,258 had KEGG homologs and 6,433 had COG homologs (Table 6).

**Table 5:** Statistics of gene information in the *D*. *cambodiana* genome.

Software	Gene Number	Gene	Average Gene	Exon	Average Exon	Intron	Average Intron
		Length (bp)					
Gene ID	53,700	109,047,509	2030.67	25,868,681	197.91	83,178,828	636.37

**Table 6:** Statistics of gene functional annotations in the *D*. *cambodiana* genome.

Annotation Database	Annotated Number (100≤Protein Length<300)	Annotated Number (Protein Length≥300)	Annotated Number (Total)	Percentage (%)
COG	2,888	1,971	6,433	11.98
GO	5,716	2,692	12,510	23.29
KEGG	4,215	2,058	9,258	17.24
KOG	5,892	3,224	13,093	24.38
Pfam	6,132	4,098	12,859	23.95
Swiss-Prot	6,832	3,853	14,873	27.70
TrEMBL	10,400	5,128	22,153	41.25
nr	10,477	5,119	22,312	41.55
nt	16,368	6,831	36,901	68.72
All	16,977	6,923	38,162	71.07

When blasted against the NCBI NR database, 22,312 (41.55%) of the 53,700 genes possessed significant similarity to plant nucleotide sequences in GenBank (Table 6). The species most represented in the NR homolog analysis were *Elaeis guineensis* and *Phoenix dactylifera*, which belong to the Arecaceae (Table 7).

**Table 7:** Top 10 hit species distribution based on Nr in the *D*. *cambodiana* genome annotation.

Species	Number	Percentage (%)
*Elaeis guineensis*	6202	27.80
*Phoenix dactylifera*	5524	24.76
*Musa acuminata*	2555	11.45
*Nelumbo nucifera*	764	3.42
*Oryza sativa*	489	2.19
*Vitis vinifera*	478	2.14
*Zea mays*	433	1.94
*Citrus sinensis*	244	1.09
*Eucalyptus grandis*	205	0.92
*Theobroma cacao*	185	0.83
Other	5233	23.46

**Fig 4:**
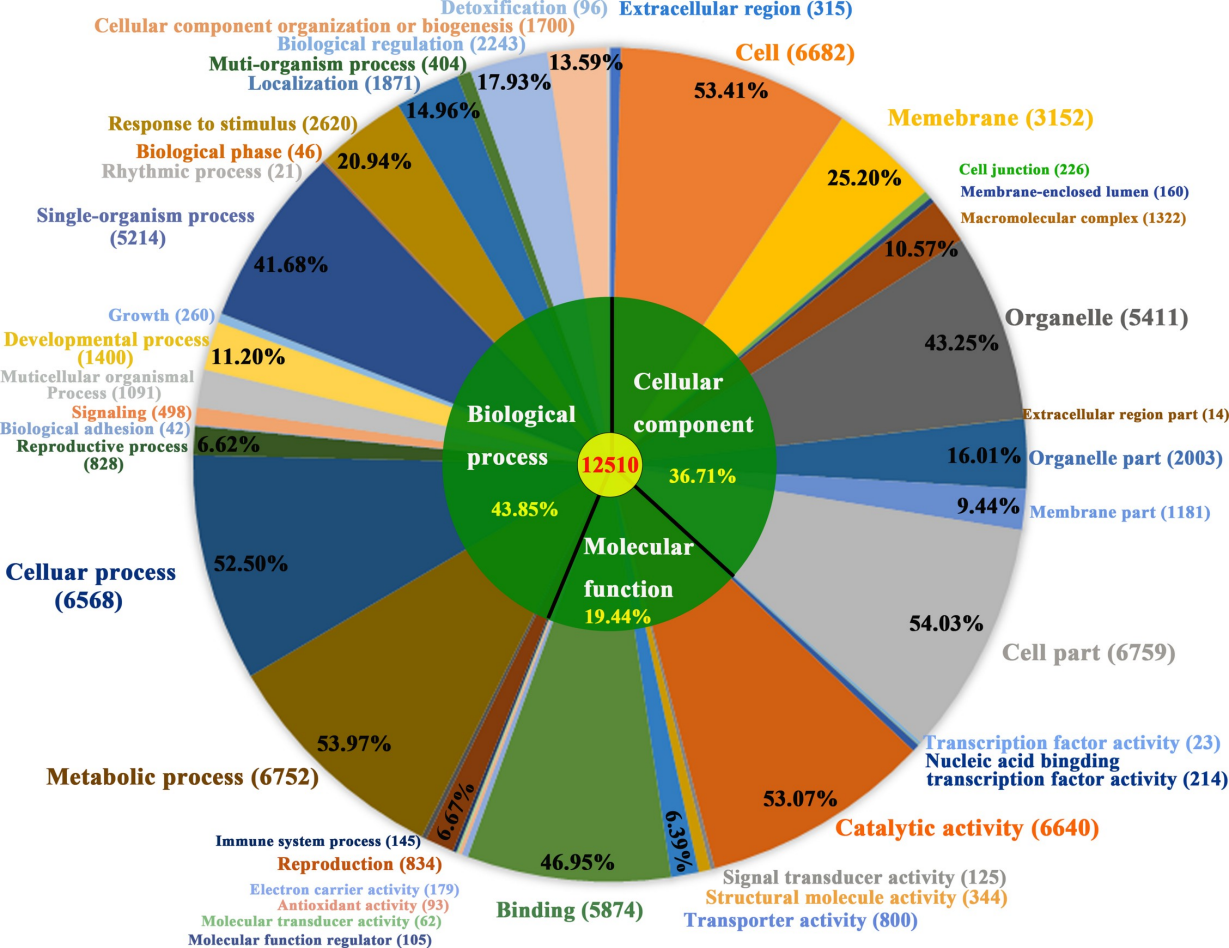
Gene Ontology classification. Genes were assigned to three categories: cellular components, molecular functions and biological process.

In total, 13,093 and 6,433 genes were matched in the KOG and COG functional classifications, respectively (Fig 4 and Table 5). The largest cluster in the KOG and COG analyses was general function prediction only (S1 and S2 Figs), followed by the posttranslational modification, protein turnover, chaperone, and signal transduction mechanism categories in KOG and the transcription (844, 9.44%), translation, ribosomal structure, and biogenesis (604, 6.75%) categories in COG.

A total of 9,258 genes were matched to 125 KEGG pathways (S4 Table). Of these, 5,698 genes (61.56%) were mapped to 94 metabolic pathways, of which 1,312 (14.17%) and 1,222 genes (13.20%) corresponded to amino acid and carbohydrate metabolism, respectively, followed by energy (850, 9.18%), lipid (420, 4.54%), glycan (607, 6.56%), nucleotide (325, 3.51%), secondary metabolite biosynthesis (267, 2.89%), cofactors and vitamins (263, 2.84%), other amino acid (248, 2.68%), and terpenoid and polyketide (184, 1.99%) metabolism. In addition, 2,492 genes (26.92%) were involved in genetic information processing, 441 genes (4.76%) with cellular processes, 355 genes (3.83%) with environment information processing, and 272 genes with organismal systems (3.93%).

**Fig 5:**
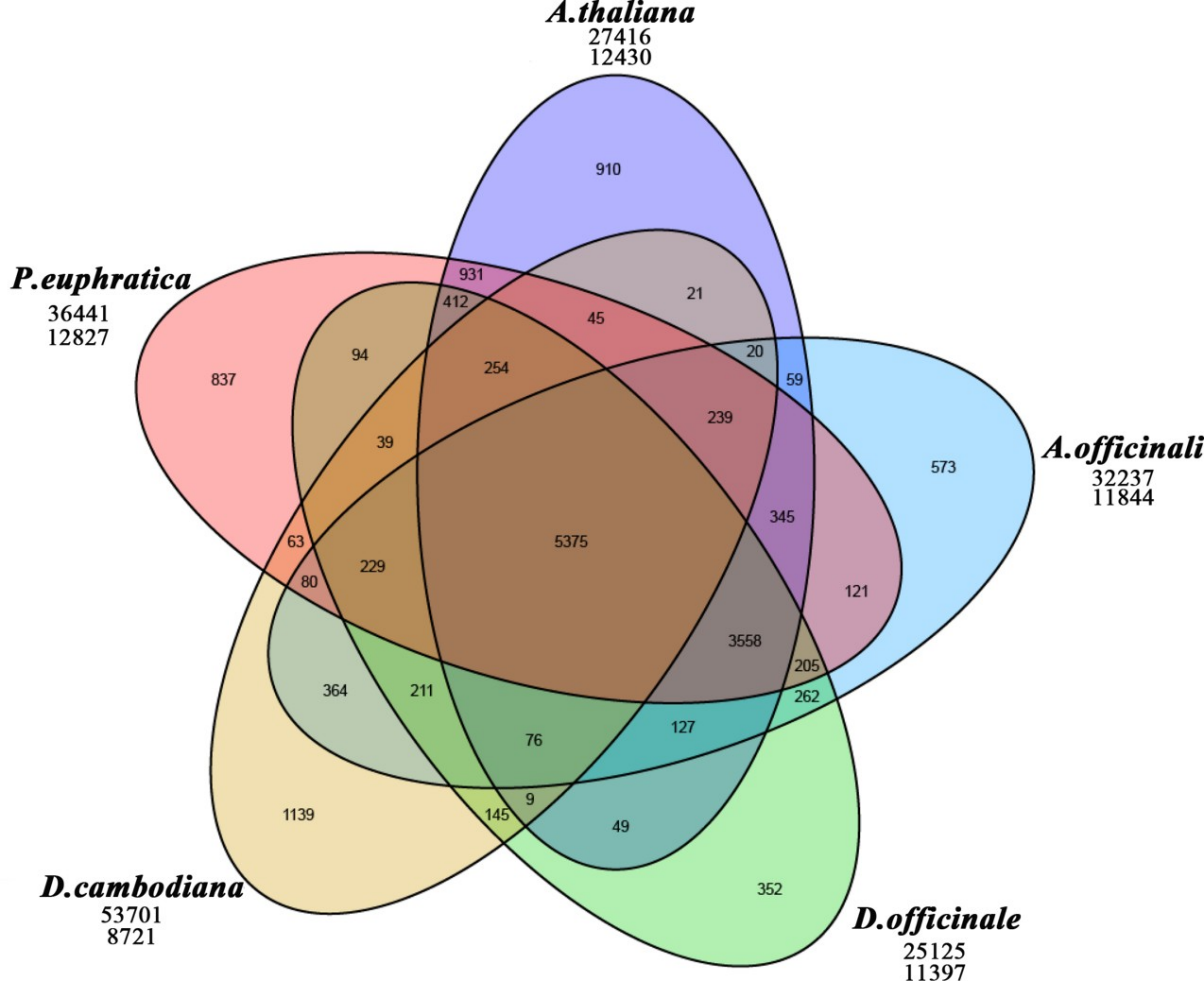
The number of gene clusters in *D*. *cambodiana* and other species. The first number under the plant species name is the total number of putative genes used for clustering. The second number under the plant species name is the number of clusters or families.

Gene family analysis revealed that 38,162 predicted family member genes in *D*. *cambodiana* were shared among five plant species. Of these, 7,582 family member genes were clustered with *Arabidopsis thaliana*, *Asparagus officinalis*, *Dendrobium officinale*, or *Populus euphratica*, whereas 1,139 predicted genes were unique to *D*. *cambodiana* (Fig 5).

### Putative genes associated with defense response during dragon’s blood formation in *D*. *cambodiana*

According to the genome annotation of *D*. *cambodiana*, many unique sequences were annotated as plant defense response genes. In this study, these unigene sequences were reanalyzed with the public RNA-seq data of dragon’s blood formation in *D*. *cambodiana*. Of these sequences, 41, 38, and 79 sequences were annotated to be involved in the synthesis of plant cell wall components, plant defense substances, and oxidative stress response, respectively (S5 Table).

**Fig 6:**
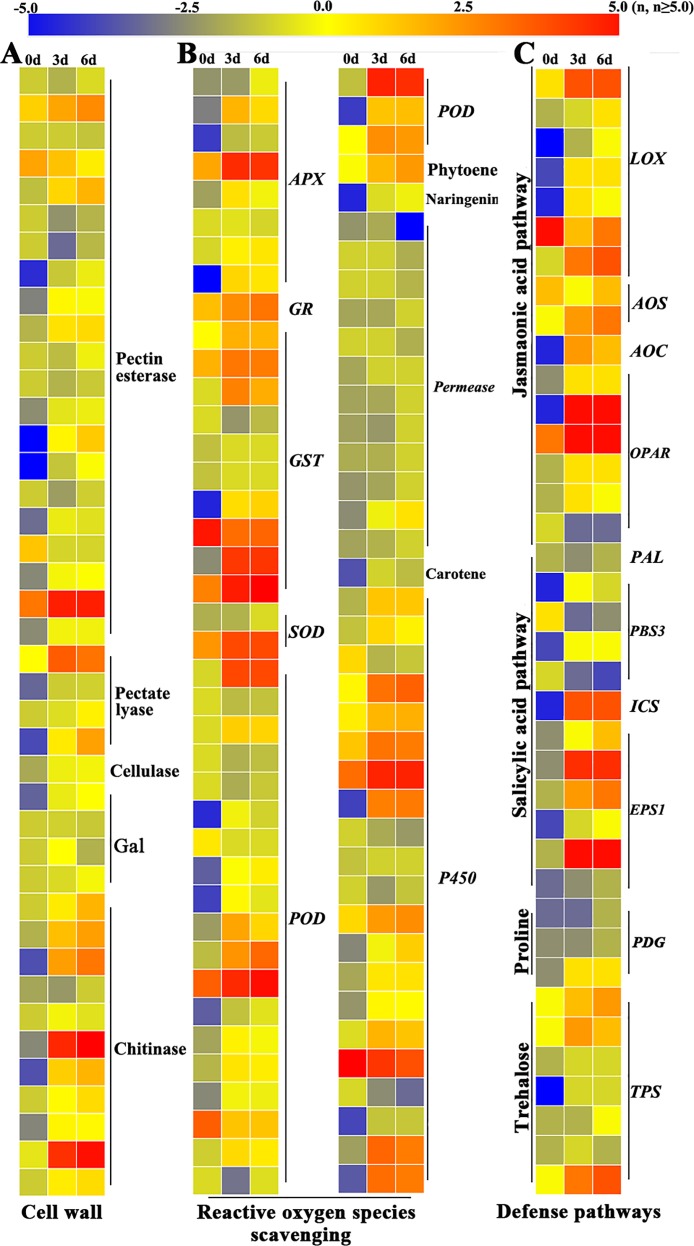
Expression profiles of genes related to defense response during dragon's blood formation in the stem of *D*. *cambodiana*. Stem samples were collected at 0, 3, and 6 days after treatment with the special inducer. Gene expression in the stems of *D*. *cambodiana* is indicated by mean-centered log_2_-transformed FPKM values, and blue, yellow and red bars show low to high expression levels. A: plant cell wall compounds synthesis related genes; B: reactive oxygen species (ROS) scavenging related genes; C: genes involved in plant defense pathways.

During the formation of dragon’s blood in *D*. *cambodiana*, one pectate lyase, two pectin esterases, and six chitinases were activated (Fig 6A). Genes encoding antioxidases (such as *APX*, *GR*, *GST*, *SOD*, and *POD*), P450 and the phytoene synthase enzyme were also upregulated (Fig 6B). Meanwhile, genes involved in plant hormone and defense compound synthesis, such as JA-related genes (*LOX*, *AOS*, *AOC*, and *OPAR*), SA-related genes (*ICS* and *EPS1*), proline-related genes (*PDG*), and trehalose-related genes (*TPS*), were also induced in this process (Fig 6C), indicating a joint effect of systemic acquired resistance and induced systemic resistance during the formation of dragon’s blood. Some other genes involved in cell wall formation, plant defense substance synthesis and oxidative stress response were simultaneously regulated during dragon’s blood formation. However, their expression was lower than that in the earlier stage of dragon’s blood formation; these genes included *PAL*, *PBS3*, and the enzymes catalyzing naringenin, permease, carotene, cellulase, or galactosidase synthesis (Fig 6).

### RT-qPCR validation of differential gene expression

**Fig 7:**
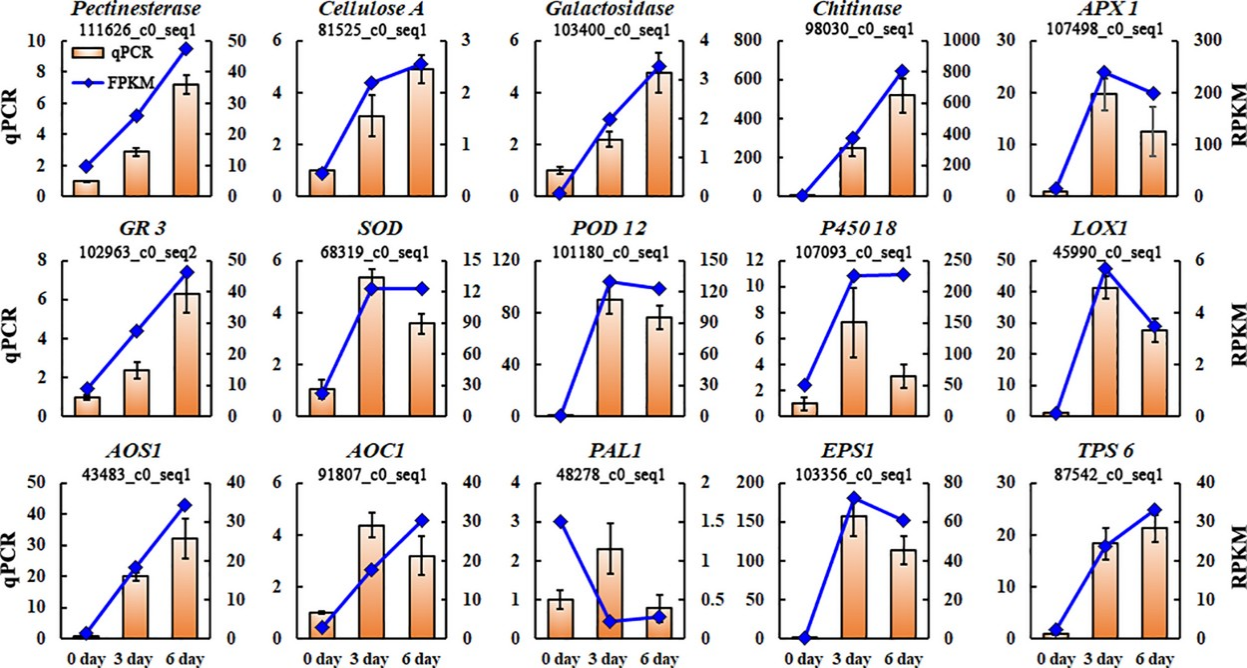
qPCR validations of 15 genes involved in plant defense response. The histograms show the qPCR results of 15 genes involved in defense response in stems of *D*.*cambodiana* after injecting the inducer in 0, 3, 6 days respectively; the line charts show the FPKM values of these unigenes. qPCR results represent the mean (±SD) of three biological replicates.

To investigate the transcriptional response of defense related genes during dragon’s blood formation were differently expression, 15 DEGs involved in plant defense response were chosen for RT-qPCR assay. Most of the selected DEGs were differentially expressed in stem under inducer treatment, showing similar patterns as reflected by FPKM values (Fig 7). Therefore, this result provided reliable and accurate transcriptional profiling data for further studies on the cross-talk between plant defense response and mechanism of dragon’s blood formation in *D*. *cambodiana*.

## Discussion

Flow cytometry and C-values evaluation were regarded as the standard or reference methods for predicting genome size before plant genome sequencing [45, 46]. Nevertheless, the development of NGS technologies has provided an affordable and effective means for obtaining the basic genomic information of non-model or emerging species [47]. Additionally, genome survey sequencing combined with k-mer analysis has been successfully applied for predicting genome size without prior information regarding the genome [48, 49]. This technique has been used in estimating the genome sizes of *Myrica rubra* [49], *Gracilariopsis lemaneiformis* [50], *Brassica juncea* [51], *Ipomoea trifida* [52], *Rosa roxburghii* [53], and *Rastrelliger kanagurta* [54]. In this study, the genome size of *D*. *cambodiana* was estimated to be 1.12–1.17 Gb based on the 21-mer distribution (Table 1). This size was within the flow cytometry prediction of 0.91–1.23 Gb [55,56].

The k-mer analysis also suggested that the GC content was between 37.35% and 39.04% (Fig 2; Table 1), which is comparable to those of *E*. *guineensis* (38.2%), *Prunus persica* (37.5%), *Pyrus bretschneideri* (37.5%), *Theobroma cacao* (34.84%), *Jatropha curcas* (34.85%), and *Hevea brasiliensis* (34.90%), but lower than those of *Selaginella moellendorffii* (45.30%) and *Malus domestica* (42.33%). A very low (< 25%) or high (> 65%) GC content may cause bias in sequencing and genome assembly and eventually affect genome analysis [57].

Medicinal or horticultural trees are generally perennial and highly heterozygous [58,59]. Previous studies have suggested that heterozygosity greater than 0.5% presents difficulties for short-read-based assembly, because a random selection learning strategy cannot be applied to heterozygous loci [60]. However, highly heterozygous genomes have been characterized using a cost-effective strategy with the Platanus software since 2015. These genomes have included those of the crown-of-thorns starfish, *Papilio glaucus*, and *Ananas comosus*, with genome heterozygosities of 0.92%, 1.8%, and 1.89%, respectively [61, 62, 63]. After a comprehensive analysis of the total data from three sequencing libraries, the heterozygosity of *D*. *cambodiana* was confirmed as 0.38%, which was lower than the threshold for a highly heterozygous genome.

SSR content is another crucial reference for the strategic selection of genome assembly. The repetitive sequence in the *D*. *cambodiana* genome was approximately 53.45% and was nearly 700 Mb in length. This ratio is lower than those in the plant genomes reported recently, including *Chenopodium quinoa* (64%) [64], *Hevea brasiliensis* (71%) [65], *Camellia sinensis* (80.9%) [66], and *Hordeum vulgare* (84%) [67].

Compared with other plant genomic information and on the basis of the GC content, heterozygosity, repetitive element content, and genome size described earlier, large-insert libraries of genomic DNA and high sequencing depth were appropriate for the whole-genome sequencing of *D*. *cambodiana*. The final genome might also be assembled under a higher k-mer value than that used in this study [64, 68]. Not only did the genome sequence survey technology provide strategies for whole-genome assembly in future projects [69], but more importantly, partial nucleic acid and protein information was simultaneously obtained via the assembly and annotation of raw reads from the genome sequence survey [53]. Such an approach could also provide more genome-level genetic information for *D*. *cambodiana* without a complete genome sequence and presumably improve the understanding of the connection between defense response and dragon’s blood formation in *D*. *cambodiana*.

In nature, only *Dracaena* trees 30–50 years of age can produce a small amount of dragon’s blood [18]. Our previous studies revealed a chemical inducer that can induce the formation of red resin in young *D*. *cambodiana* [18, 19]. This special inducer, which contains 37.5 g/L NaCl and 1.25 ml/L acetic acid, can quickly induce the formation of the major constituents of dragon’s blood in the stems of 3-year-old *D*. *cambodiana*; in particular, flavonoids can be detected by HPLC analysis at 3 days, 6 days and 9 days after injecting the inducer. After inducer injection treatment for 6–12 months, qualified red resin can be collected from the stems of *D*. *cambodiana*. Previous studies have also reported that red resin production can be induced in healthy stems by the pathogenic microbes, such as *Fusarium graminearum* and *Fusarium proliferatum*, that were isolated from a dragon’s blood–secreting stem of *D*. *cochinchinensis* [4, 14]. Plant defense responses may be involved in dragon’s blood formation in *Dracaena* species, based on the phenomena of wounding, induction, microbial infection, and red resin formation.

A common feature of microbial infection in plants is passing though the plant cell wall, which is the first barrier of plants against pathogen attack [70]. To this end, the main structural components of plant cell walls, such as pectin, cellulose, chitin, and other polysaccharides, are depolymerized with special enzymes secreted by the microbe [71]. In this process, the plant genes encoding the enzymes to degrade these components can also be regulated by the pathogen [72]. Subsequent studies have found that such genes are a part of the plant immune system and can be regulated by various stresses [73]. The gene expression profiles examined in this study indicated that a chemical inducer can regulate genes encoding galactosidase, cellulase, chitinase, pectin esterase, and lyase in *D*. *cambodiana* (Fig 6A).

All plant stress reactions can produce ROS, which are an important signaling factor in plants and may link to systemic acquired resistance, programmed cell death, and plant hormone signaling [74–76]. However, the presence of ROS can further injure healthy plant cells. Hence, the antioxidant system is triggered during the ROS burst to protect normal cells from superfluous ROS damage. The antioxidant system commonly includes such antioxidases as SOD, POD, and GST [77–80]. Here, the expression patterns of *APX*, *GR*, *GST*, *POD*, permease, and *P450s* were upregulated during dragon’s blood formation in *D*. *cambodiana*. Furthermore, some genes expressed during dragon’s blood biosynthesis encoded natural antioxidants [81, 82]. For example, phytoene and naringenin expression was altered in the stems of *D*. *cambodiana* when it was injected with a chemical inducer (Fig 6B). This finding indirectly suggested that a defense signal was released during dragon’s blood formation in *D*. *cambodiana*; however, the detailed mechanism should be further investigated.

Plant hormones play important roles in regulating plant development and defense response. Genes related to plant development and defense response can be regulated by pathogens, insects, wounding, exogenous JA, SA, benzothiadiazole (BTH), ABA, NaCl, and other biotic and abiotic stresses [16, 17, 83–85]. In this study, the expression of JA-related genes (*LOX*, *AOS*, *AOC*, and *OPAR*), SA-related genes (*PAL*, *PBS3*, *ICS*, and *EPS1*), and osmosis-related genes (*PDG* and *TPS*) in the stems of *D*. *cambodiana* was enhanced by a chemical inducer (Fig 6C). Previous studies have indicated that plant hormones, which are essential for plant response to biotic and abiotic stresses, can modulate secondary metabolite accumulation in plants [16, 84, 86]. Consistent with previous reports [18, 19], this study further demonstrated the potential connection between the defense response and dragon’s blood formation in *D*. *cambodiana*.

## Conclusions

This study is the first to report the genomic characterization of *Dracaena* on a genome-wide scale. Of the 50 *Dracaena* species, *D*. *cambodiana* is one of the most important in terms of its horticultural and medicinal values in China and Southeast Asia. However, limited genetic information has impeded studies of *D*. *cambodiana*, especially the mechanism of dragon’s blood formation. The regulatory expression analysis of candidate genes involved in the plant defense response may help elucidate this mechanism in *D*. *cambodiana*. The 53,700 total genes derived from our assembly may facilitate genetic and genomic studies. The characterization of this genetic information may provide fundamental parameters for the sequencing and assembly strategies of the *D*. *cambodiana* genome program.

## Supporting information

S1 Fig Gene assignment to KOG functional classifications in *D. cambodiana*. (TIF)

S2 Fig Gene assignment to COG functional classifications in *D. Cambodian*. (TIF)

S1 Table Sequences of specific primers used for real-time quantitative PCR. (XLSX)

S2 Table Occurrence of SSR motifs in the genome survey of *D. cambodiana*. (XLS)

S3 Table Interactive supporting of predicted genes and transcripts in public RNA-seq. (XLSX)

S4 Table Number of genes of *D. cambodiana* mapped to KEGG pathways. (PDF)

S5 Table The expression profiles (RPKM) of defense related genes after stem of *Dracaena cambodiana* injected by inducer. (XLSX)
