# Supplementary figures and images for: Genome survey sequencing for the characterization of genetic background of Dracaena cambodiana and its defense response during dragon’s blood formation
Source: PLoS One. 2018 Dec 14;13(12):e0209258. doi: 10.1371/journal.pone.0209258 (PMC6294377; doi:10.1371/journal.pone.0209258)

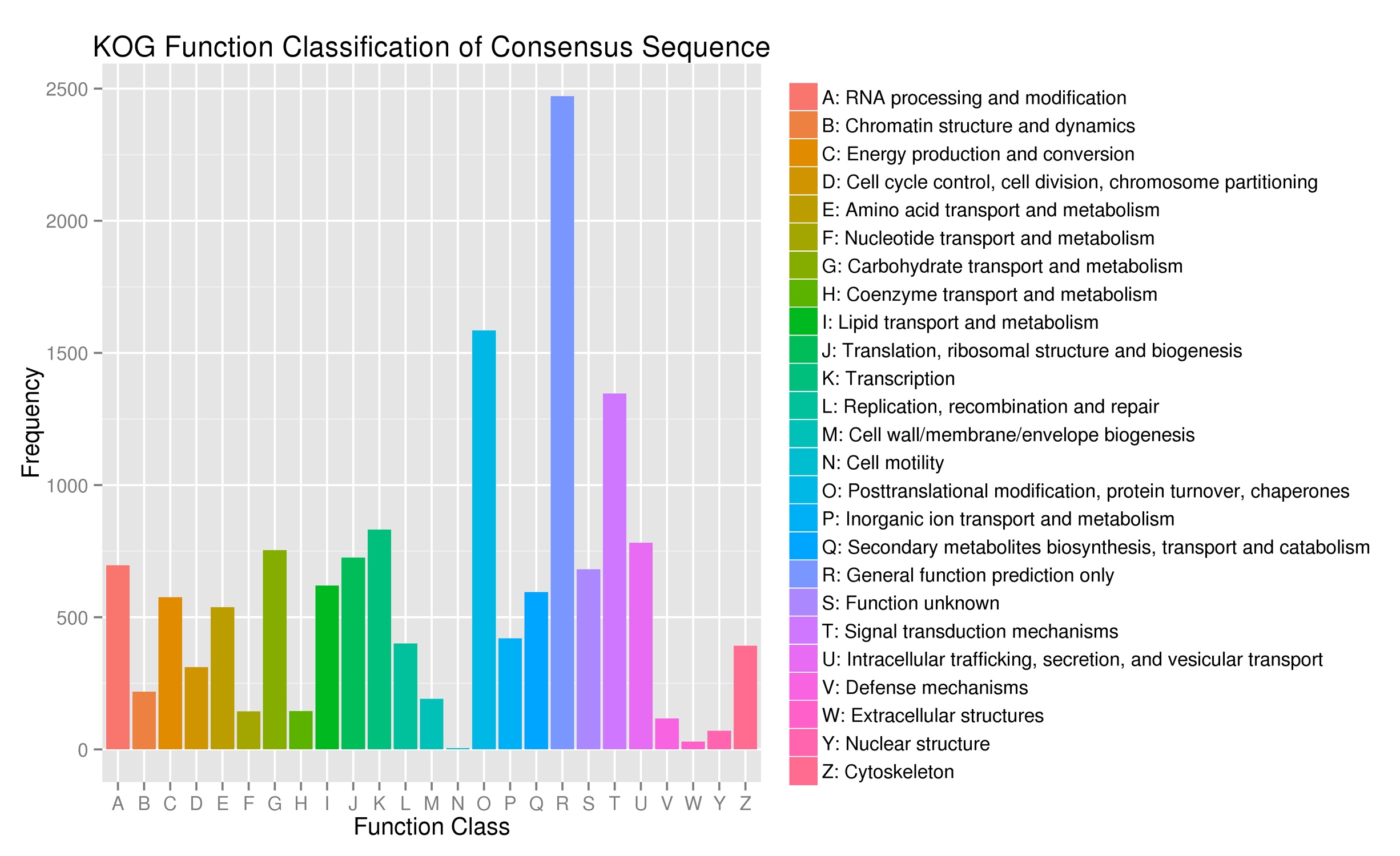

Supplement: S1 Fig — (TIF) [file pone.0209258.s001.tif]

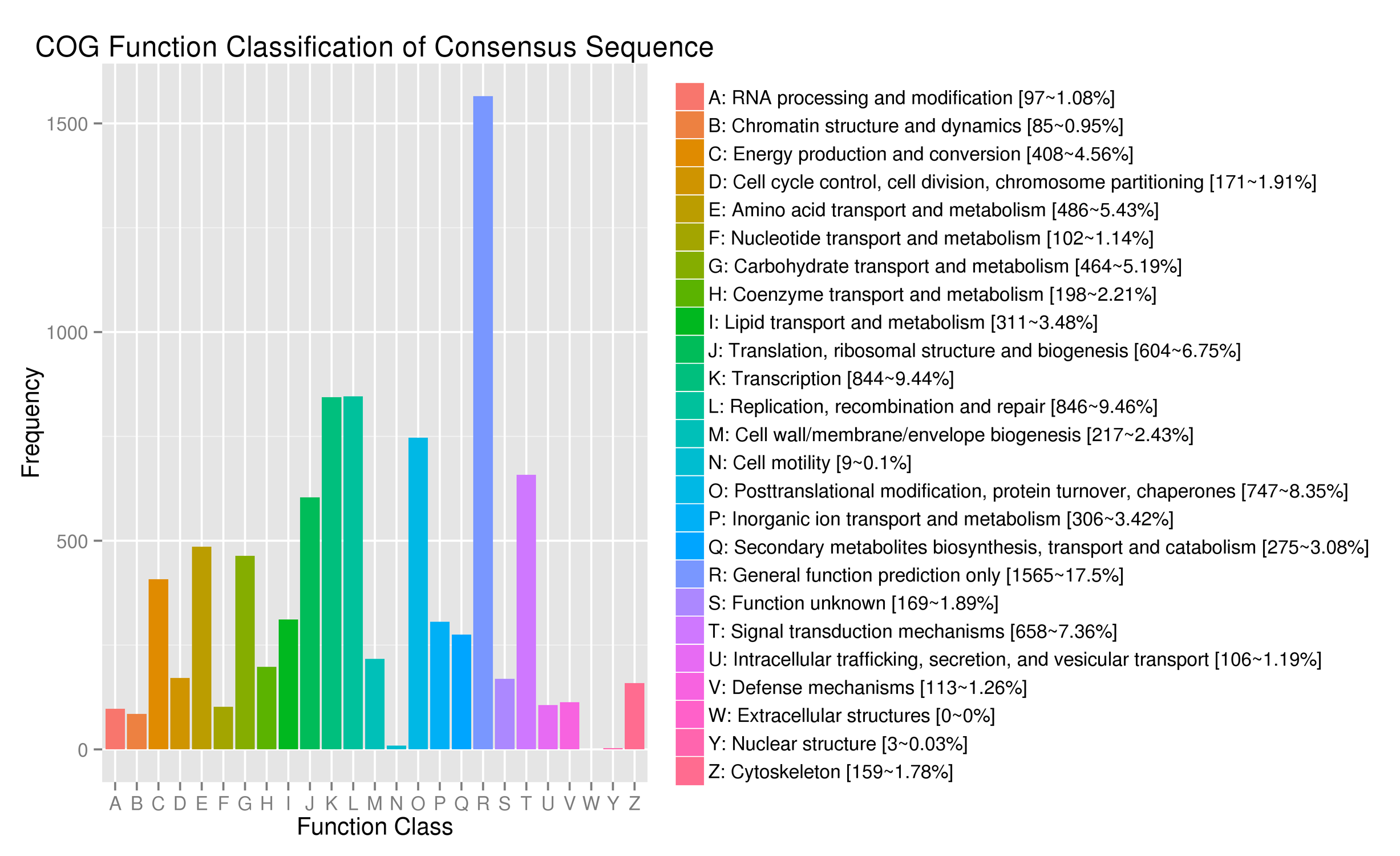

Supplement: S2 Fig — (TIF) [file pone.0209258.s002.tif]
