# Supplementary material for: Genome survey sequencing for the characterization of genetic background of Dracaena cambodiana and its defense response during dragon’s blood formation
Source: PLoS One. 2018 Dec 14;13(12):e0209258. doi: 10.1371/journal.pone.0209258 (PMC6294377; doi:10.1371/journal.pone.0209258)
Supplement: S4 Table — (PDF) [file pone.0209258.s006.pdf]

S4 Table. Number of genes of *D.cambodiana* mapped onto KEGG pathways

Pathway		Pathway Id	Gene Number
Carbohydrate metabolism (1222,13.20%)	Glycolysis / Gluconeogenesis	ko00010	178
	Citrate cycle (TCA cycle)	ko00020	91
	Pentose phosphate pathway	ko00030	81
	Pentose and glucuronate interconversions	ko00040	84
	Fructose and mannose metabolism	ko00051	92
	Galactose metabolism	ko00052	84
	Ascorbate and aldarate metabolism	ko00053	50
	Pyruvate metabolism	ko00620	147
	Glyoxylate and dicarboxylate metabolism	ko00630	78
	Propanoate metabolism	ko00640	58
	Butanoate metabolism	ko00650	28
	C5-Branched dibasic acid metabolism	ko00660	9
	Starch and sucrose metabolism	ko00500	242
Energy metabolism (850, 9.18%)	Nitrogen metabolism	ko00910	38
	Sulfur metabolism	ko00920	41
	Oxidative phosphorylation	ko00190	194
	Photosynthesis	ko00195	88
	Carbon fixation in photosynthetic organisms	ko00710	103
	Photosynthesis - antenna proteins	ko00196	15
	Carbon metabolism	ko01200	371
Lipid metabolism (420, 4.54%)	Fatty acid biosynthesis	ko00061	81
	Fatty acid elongation in mitochondria	ko00062	29
	Fatty acid metabolism	ko00071	62
	Synthesis and degradation of ketone bodies	ko00072	9
	Steroid biosynthesis	ko00100	44
	Biosynthesis of unsaturated fatty acids	ko01040	30
	Ether lipid metabolism	ko00565	45
	Arachidonic acid metabolism	ko00590	22
	Linoleic acid metabolism	ko00591	17
	alpha-Linolenic acid metabolism	ko00592	37
	Sphingolipid metabolism	ko00600	44
Nucleotide metabolism (325, 3.51%)	Purine metabolism	ko00230	179
	Pyrimidine metabolism	ko00240	146
Amino acid metabolism (1312, 14.17%)	Alanine, aspartate and glutamate metabolism	ko00250	67
	Glycine, serine and threonine metabolism	ko00260	93
	Cysteine and methionine metabolism	ko00270	106

	Valine, leucine and isoleucine degradation	ko00280	58
	Valine, leucine and isoleucine biosynthesis	ko00290	22
	Lysine biosynthesis	ko00300	28
	Lysine degradation	ko00310	48
	Arginine and proline metabolism	ko00330	84
	Histidine metabolism	ko00340	22
	Tyrosine metabolism	ko00350	53
	Phenylalanine metabolism	ko00360	108
	Tryptophan metabolism	ko00380	34
	Phenylalanine, tyrosine and tryptophan biosynthesis	ko00400	56
	2-Oxocarboxylic acid metabolism	ko01210	70
	Fatty acid metabolism	ko01212	112
	Biosynthesis of amino acids	ko01230	351
Metabolism of other amino acids (248, 2.68%)	beta-Alanine metabolism	ko00410	60
	Taurine and hypotaurine metabolism	ko00430	10
	Selenocompound metabolism	ko00450	36
	Cyanoamino acid metabolism	ko00460	42
	Glutathione metabolism	ko00480	100
Glycan biosynthesis, metabolism (607, 6.56%)	N-Glycan biosynthesis	ko00510	46
	Other glycan degradation	ko00511	34
	Other types of O-glycan biosynthesis	ko00514	7
	Amino sugar and nucleotide sugar metabolism	ko00520	158
	Polyketide sugar unit biosynthesis	ko00523	3
	Glycosaminoglycan degradation	ko00531	17
	Glycerolipid metabolism	ko00561	104
	Inositol phosphate metabolism	ko00562	76
	Glycosylphosphatidylinositol(GPI)-anchor biosynthesis	ko00563	12
	Glycerophospholipid metabolism	ko00564	125
	Glycosphingolipid biosynthesis - globo series	ko00603	16
	Glycosphingolipid biosynthesis - ganglio series	ko00604	9
Cofactors and vitamins metabolism (263, 2.84%)	One carbon pool by folate	ko00670	21
	Thiamine metabolism	ko00730	16
	Riboflavin metabolism	ko00740	12
	Vitamin B6 metabolism	ko00750	17
	Nicotinate and nicotinamide metabolism	ko00760	26
	Pantothenate and CoA biosynthesis	ko00770	26
	Biotin metabolism	ko00780	23
	Lipoic acid metabolism	ko00785	7
	Folate biosynthesis	ko00790	16

	Porphyrin and chlorophyll metabolism	ko00860	53
	Ubiquinone and other terpenoid-quinone biosynthesis	ko00130	46
Terpenoids and polyketides metabolism (184, 1.99%)	Terpenoid backbone biosynthesis	ko00900	60
	Monoterpenoid biosynthesis	ko00902	4
	Limonene and pinene degradation	ko00903	3
	Diterpenoid biosynthesis	ko00904	24
	Brassinosteroid biosynthesis	ko00905	10
	Carotenoid biosynthesis	ko00906	42
	Zeatin biosynthesis	ko00908	28
	Sesquiterpenoid and triterpenoid biosynthesis	ko00909	13
Secondary metabolites Biosynthesis (267, 2.89%)	Caffeine metabolism	ko00232	3
	Phenylpropanoid biosynthesis	ko00940	126
	Flavonoid biosynthesis	ko00941	45
	Flavone and flavonol biosynthesis	ko00944	1
	Stilbenoid, diarylheptanoid and gingerol biosynthesis	ko00945	11
	Isoquinoline alkaloid biosynthesis	ko00950	31
	Tropane, piperidine and pyridine alkaloid biosynthesis	ko00960	39
	Glucosinolate biosynthesis	ko00966	1
	Degradation of aromatic compounds	ko01220	10
Genetic Information Processing (2492, 26.92%)	Spliceosome	Ko03040	295
	Ribosome	ko03010	287
	Aminoacyl-tRNA biosynthesis	ko00970	99
	RNA transport	ko03013	266
	Protein processing in endoplasmic reticulum	ko04141	295
	Ubiquitin mediated proteolysis	ko04120	154
	Ribosome biogenesis in eukaryotes	ko03008	122
	mRNA surveillance pathway	ko03015	172
	RNA degradation	ko03018	169
	Proteasome	ko03050	89
	Nucleotide excision repair	ko03420	76
	Protein export	ko03060	60
	Basal transcription factors	ko03022	58
	DNA replication	ko03030	62
	Homologous recombination	ko03440	66
	Mismatch repair	ko03430	50
	RNA polymerase	ko03020	48
	Base excision repair	ko03410	53
	SNARE interactions in vesicular transport	ko04130	44
	Sulfur relay system	ko04122	12

	Non-homologous end-joining	ko03450	15
Environmental Information Processing (355, 3.83%)	Vancomycin resistance	Ko01502	5
	Plant hormone signal transduction	ko04075	224
	Phosphatidylinositol signaling system	ko04070	78
	ABC transporters	ko02010	48
Cellular processes (441, 4.76%)	Endocytosis	Ko04144	190
	Phagosome	ko04145	107
	Peroxisome	ko04146	100
	Regulation of autophagy	ko04140	44
Organismal Systems (272, 2.93%)	Circadian rhythm-plant	Ko04712	57
	Plant-pathogen interaction	ko04626	215
